# Modern aspects of the use of natural polyphenols in tumor prevention and therapy

**DOI:** 10.3389/fcell.2022.1011435

**Published:** 2022-09-12

**Authors:** Galina Sufianova, Ilgiz Gareev, Ozal Beylerli, Jianing Wu, Alina Shumadalova, Albert Sufianov, Xin Chen, Shiguang Zhao

**Affiliations:** ^1^ Department of Pharmacology, Tyumen State Medical University, Tyumen, Russia; ^2^ Educational and Scientific Institute of Neurosurgery, Peoples’ Friendship University of Russia (RUDN University), Moscow, Russia; ^3^ Department of Neurosurgery, Shenzhen University General Hospital, Shenzhen, China; ^4^ Department of General Chemistry, Bashkir State Medical University, Ufa, Russia; ^5^ Department of Neurosurgery, Sechenov First Moscow State Medical University (Sechenov University), Moscow, Russia; ^6^ Department of Neurosurgical Laboratory, The First Affiliated Hospital of Harbin Medical University, Harbin, China

**Keywords:** polyphenols, flavonoids, anti-tumor therapy, prevention, signal pathways, oncogenesis

## Abstract

Polyphenols are secondary plant metabolites or organic compounds synthesized by them. In other words, these are molecules that are found in plants. Due to the wide variety of polyphenols and the plants in which they are found, these compounds are divided according to the source of origin, the function of the polyphenols, and their chemical structure; where the main ones are flavonoids. All the beneficial properties of polyphenols have not yet been studied, since this group of substances is very extensive and diverse. However, most polyphenols are known to be powerful antioxidants and have anti-inflammatory effects. Polyphenols help fight cell damage caused by free radicals and immune system components. In particular, polyphenols are credited with a preventive effect that helps protect the body from certain forms of cancer. The onset and progression of tumors may be related directly to oxidative stress, or inflammation. These processes can increase the amount of DNA damage and lead to loss of control over cell division. A number of studies have shown that oxidative stress uncontrolled by antioxidants or an uncontrolled and prolonged inflammatory process increases the risk of developing sarcoma, melanoma, and breast, lung, liver, and prostate cancer. Therefore, a more in-depth study of the effect of polyphenolic compounds on certain signaling pathways that determine the complex cascade of oncogenesis is a promising direction in the search for new methods for the prevention and treatment of tumors.

## 1 Introduction

**FIGURE 1:**
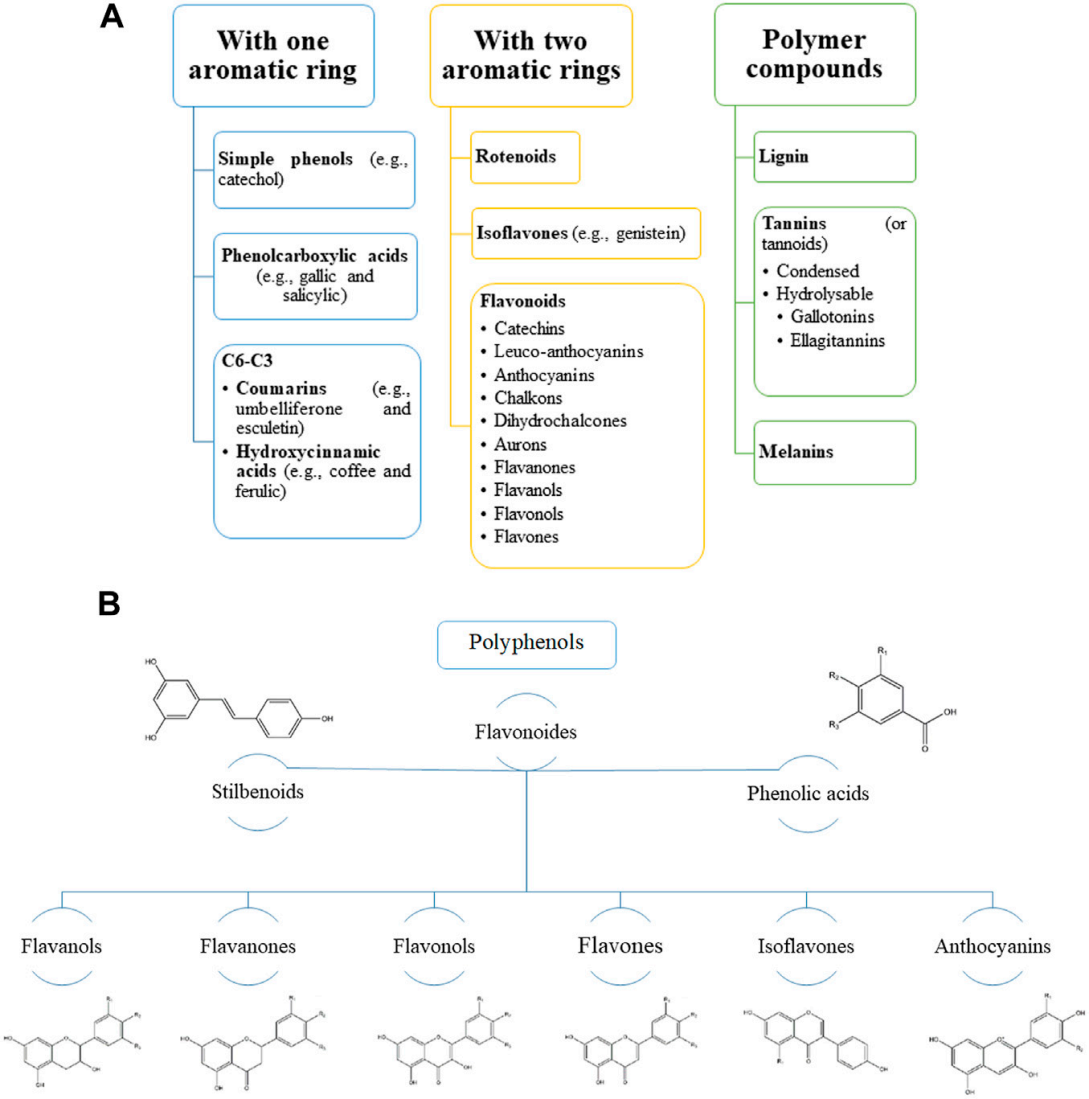
Main groups of natural polyphenolic compounds **(A–B)**. **(A)** Phenolic compounds (polyphenols) constitute one of the most numerous and widespread groups of plant substances. The structure of polyphenols can contain both simple molecules (phenolic acids) and highly polymerized compounds (condensed tannins). **(B)** It should be noted that the main share here falls on flavonoids. Structural changes in the rings subdivide flavonoids into several families: flavonols, flavones, flavanols, isoflavones, anthocyanins, etc. These families are often found in the form of glycosides. Various variations of flavonoids are linked by a common biosynthesis.

Polyphenols are naturally occurring compounds found primarily in fruits, vegetables, cereals, beverages, and dry beans. Polyphenols are secondary metabolites of plants and are usually involved in protection against ultraviolet (UV) radiation or against aggression from pathogens (Figure 1) (Singla et al., 2019; Reed and de Freitas, 2020). In food, polyphenols can contribute bitterness, astringency, color, taste, odor, and oxidative stability. Animal, human, and epidemiological studies indicate that various polyphenols have antioxidant and anti-inflammatory properties that may have preventive and/or therapeutic effects in cardiovascular disease, neurodegenerative disorders, fat metabolism disorders, and tumors (Yahfoufi et al., 2018; Potì et al., 2019). In recent years, interest in polyphenols has increased as they are the subject of scientific research due to their possible beneficial effects on human health. Also, the main advantage of polyphenolic compounds is their use as chemoprophylaxis due to low toxicity and high tolerability (Singla et al., 2019; Reed and de Freitas, 2020).

The main indicator in the use of polyphenols is bioavailability. Bioavailability is the proportion of a nutrient that is digested, absorbed, and metabolized through normal pathways. The bioavailability of each polyphenol is different, but their relationship between the amount of polyphenols in food and their bioavailability in the human body is not fully understood. It is important that the chemical structure of polyphenols, and not their concentration, determines the rate and extent of absorption, as well as the nature of the metabolites circulating in plasma. The most abundant polyphenols in the diet are not necessarily those that show the highest concentration of active metabolites in target tissues; therefore, the biological properties of polyphenols differ significantly from one polyphenol to another (Brglez Mojzer et al., 2016; Rogovskii, 2022). Evidence from preclinical and clinical studies indicates that the antioxidant and anti-inflammatory properties of polyphenols may potentially prevent or treat various tumors (Table 1) (Henning et al., 2015; Gee et al., 2017; Silva et al., 2019; Zeng et al., 2019; Basak et al., 2020; Lee et al., 2021; Mallet et al., 2021; Rojas-Ochoa et al., 2021; Shan et al., 2021; Wang et al., 2021). This review focuses on the current understanding of the biological effects of polyphenolic compounds and their importance in health and tumor prevention and therapy.

**TABLE 1:** Results of some preclinical and clinical experiments on the study of the therapeutic effect of polyphenols in tumors.

Polyphenol	Tumor type	Study model	Gene-targets	Mechanism of action	References
PEBP	Breast Cancer	*In vitro*	FOXO1 and miR-145	Inhibits breast cancer development and progression	Mallet et al. (2021)
EGCG	TNBC	*In vitro* and *in vivo*	PRODH and alpha-SMA	Sensitizes TNBC tumor cells to clinical therapeutic drugs. Inhibits tumor growth and prevents tumor cell metastasis	Lee et al. (2021)
APG-157	Oral cancer	A double-blind, randomized, placebo-controlled, phase I clinical trial	IL-1β, IL-6, and IL-8	Increases expression of genes associated with differentiation and T-cell recruitment to the tumor microenvironment	Basak et al. (2020)
Polyphenols of green and black tea	Prostate cancer	Open label, randomized, phase II trial	Ki67, apoptosis Bcl-2, Bax, Tunel, NF-κB, and 8OHdG	Anti-inflammatory effect. Promotes tumor cells death. Decrease in serum PSA	Henning et al. (2015)
Polyphenon E [a green tea polyphenol formulation primarily consisting of EGCG]	Bladder cancer	Multi-centered, randomized, double-blind, placebo-controlled, phase II trial	PCNA, MMP-2, clusterin, VEGF, p27, IGF-1, IGFBP-3	Inhibits invasion, angiogenesis, tumor cells migration, and progression	Gee et al. (2017)
AVP	Glioblastoma	*In vitro*	NF-κB, IκB-α, TNF-α, TRAIL, caspase-3 and caspase-9	Inhibitory effect on glioma cells: inhibits proliferation and enhances tumor cell apoptosis	Zeng et al. (2019)
Curcumin	Glioblastoma	*In vitro*	ERK/MAPK pathway	Inhibits adverse psychological stress‐induced proliferation and invasion of tumor cells	Wang et al. (2021)
NDGA	Medulloblastoma	*In vitro*	Glutathione/glutathione disulfide	Induce oxidative stress, G2/M and S-G2/M cell cycle arrest, and tumor cells apoptosis	Rojas-Ochoa et al. (2021)
Polyphenol complex catechin:lysine 1:2	Breast, pancreatic and colorectal cancer	*In vitro*	JAK2/STAT3 and Wnt pathway	Antimigratory and pro-apoptotic effects	Silva et al. (2019)
BPIS	Colorectal cancer	*In vitro*	Akt, Cyclin B1, CDK1, and miR-149	Increases the chemosensitivity, induce cell cycle arrest in G2/M phase	Shan et al. (2021)

TNBCs, triple-negative breast cancers; PEBP, polyphenol enriched blueberry preparation; EGCG, epigallocatechin-3-gallate; AVP, apocynum venetum polyphenol; NDGA, polyphenols α-mangostin and nordihydroguaiaretic acid; BPIS, polyphenol from foxtail millet bran; FOXO1, forkhead box protein O1; PRODH, proline dehydrogenase; alpha-SMA, smooth muscle alpha-actin; IL-1β, 6, 8, interleukin -1β, 6, 8; Ki-67, tissue immunostaining of proliferation apoptosis; Bcl-2, B-cell lymphoma 2; Bax, Bcl-2-associated X protein; NF-κB, nuclear and cytoplasmic nuclear factor kappa B; 8OHdG, 8-hydroxydeoxy-guanosine; PCNA, proliferating cell nuclear antigen; MMP-2, matrix metalloproteinase-2; VEGF, vascular endothelial growth factor; IGF-1, insulin-like growth factor 1; IGFBP-3, insulin-like growth factor binding protein-3; IκB-α, nuclear factor of kappa light polypeptide gene enhancer in B-cells inhibitor, alpha; TNF-α, tumor necrosis factor-alpha; TRAIL, tumor necrosis factor ligand superfamily member 10; ERK, extracellular signal-regulated kinase; MAPK, mitogen-activated protein kinase; JAK2, janus kinase 2; STAT3, signal transducer and activator of transcription 3; CDK1, cyclin-dependent kinase 1; PSA, prostate-specific antigen.

## 2 The effectiveness of polyphenols

In Table 1, various types of tumors are reviewed for which polyphenolic compounds have been used as therapy and have been positive. From the indicators in Table 1, the dominant explanation for these benefits is the neutralization of free radicals, the formation of stabilized chemical complexes, thus preventing further reactions. There is also evidence of an additional mechanism by which polyphenols protect against oxidative stress by producing hydrogen peroxide (H2O2), which may then help regulate immune response actions such as cell growth. It is believed that polyphenols have an anticarcinogenic effect, inhibit cell growth, causing aging or apoptosis of tumor cells, and their differential redox status can selectively affect tumor cells.

The effect of polyphenols on tumor cells is most often protective. To date, many polyphenols, such as quercetin, catechins, isoflavones, lignans, flavanones, ellagic acid, red wine polyphenols, resveratrol, and curcumin, have been studied and tested for effectiveness (Brglez Mojzer et al., 2016; Rogovskii, 2022). All of them showed protective effects in experimental models with tumors, their mechanisms of action turned out to be different.

Oncogenesis is a multistage and microevolutionary process. There are three main stages of oncogenesis: initiation, promotion and progression. Initiated cells thus may have the ability to transform into a malignant tumor if further development follows. Development is influenced by factors that do not alter the DNA sequence and include the selection of initiated cells (Patterson et al., 2018; Peters and Gonzalez, 2018).

**FIGURE 2:**
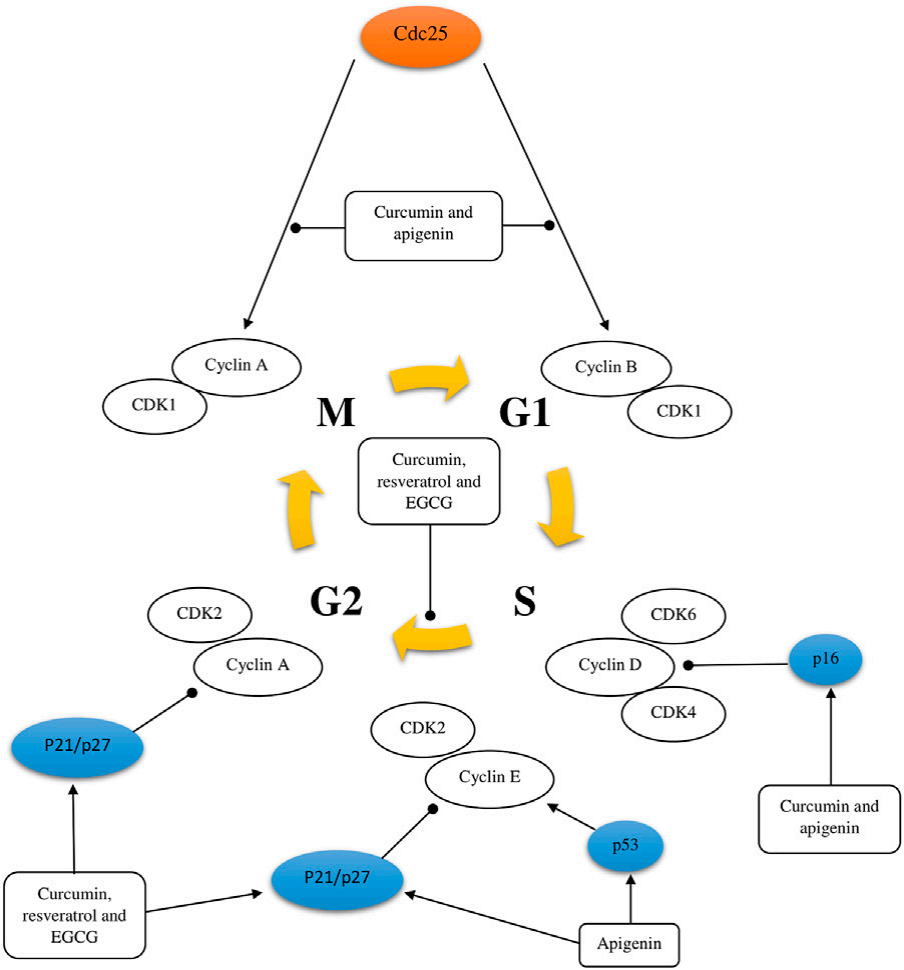
Schematic representation of cell cycle regulation. The activating and inhibitory effect of some polyphenols [curcumin, resveratrol, apigenin, and epigallocatechin-3-gallate (EGCG)] on the cell cycle of a tumor cell was shown.

**FIGURE 3:**
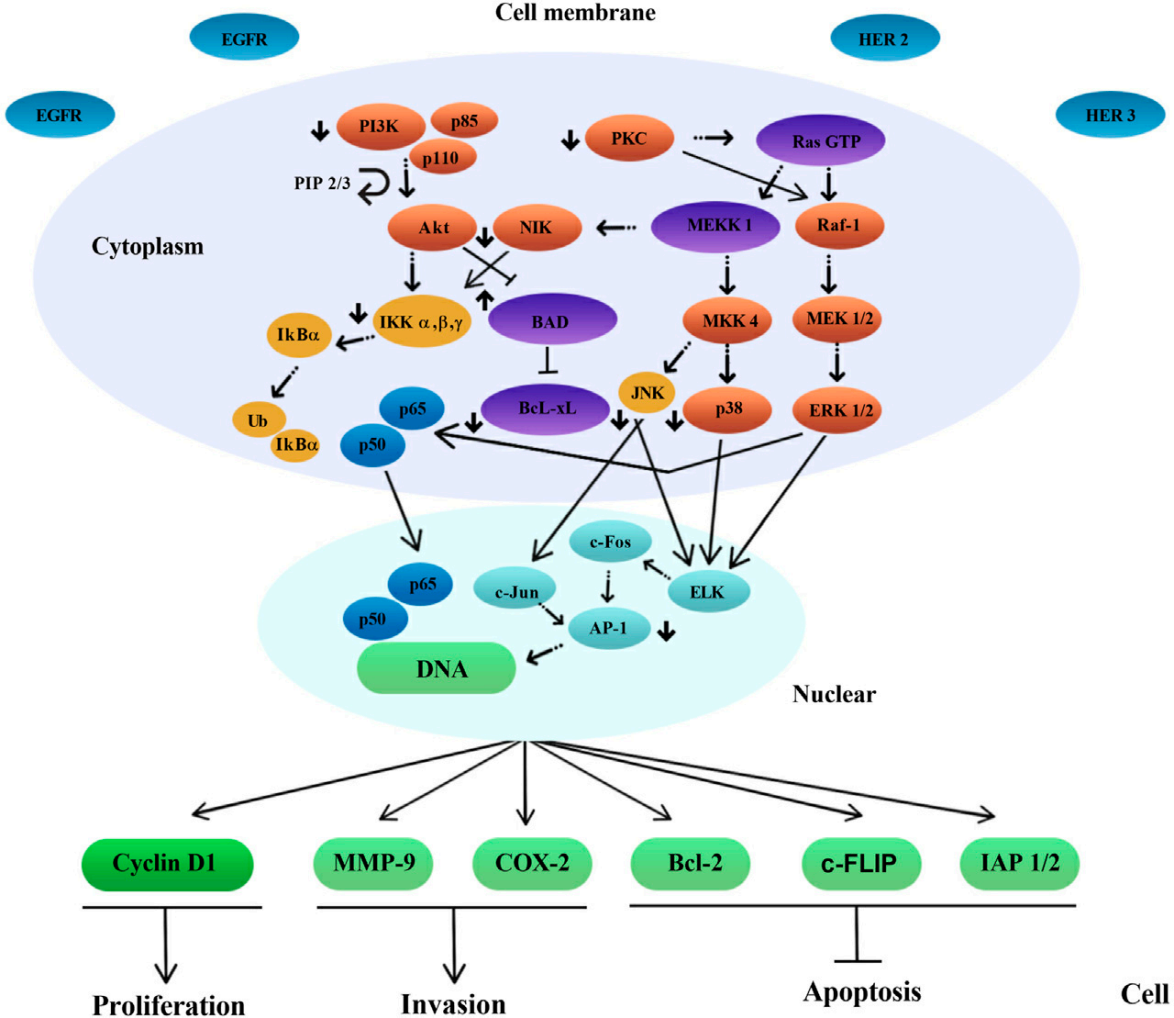
A scheme of intracellular signal transduction is presented. The inhibitory effect of epigallocatechin-3-gallate (EGCG) on some protein kinase signaling pathways and transcription factors has been shown, as a complex of p65 and p50 subunits. Note: EGFR, Epidermal growth factor receptor; HER 2/3, Human epidermal growth factor receptor 2/3; PI3K, Phosphoinositide 3-kinases; PKC, Protein kinase C; Ras GTP, RAS guanosine triphosphate; NIK, NF-kappa-B-inducing kinase; MKK4, Mitogen-activated protein kinase kinase 4; MEK 1/2, Mitogen-activated protein kinase kinase 1/2; ERK 1/2, Extracellular signal-regulated kinase-1/2; JNK, c-Jun N-terminal Kinase; BAD, BCL2-associated agonist of cell death; PIP 2/3, Prolactin induced protein 2/3; Bcl-xL, B-cell lymphoma-extra large; IKK α, β, γ, Cytokine-responsive IkappaB kinase α, β, γ; IkBα, Nuclear factor of kappa light polypeptide gene enhancer in B-cells inhibitor, alpha; Ub, Ubiquitin B; AP-1, Activator protein 1; MMP-9, Matrix metallopeptidase 9; COX-2, cyclooxygenase 2; Bcl-2, B-cell lymphoma-2; c-FLIP, Cellular FLICE-like inhibitory protein; IAP 1/2, Inhibitor of apoptosis 1/2.

**FIGURE 4:**
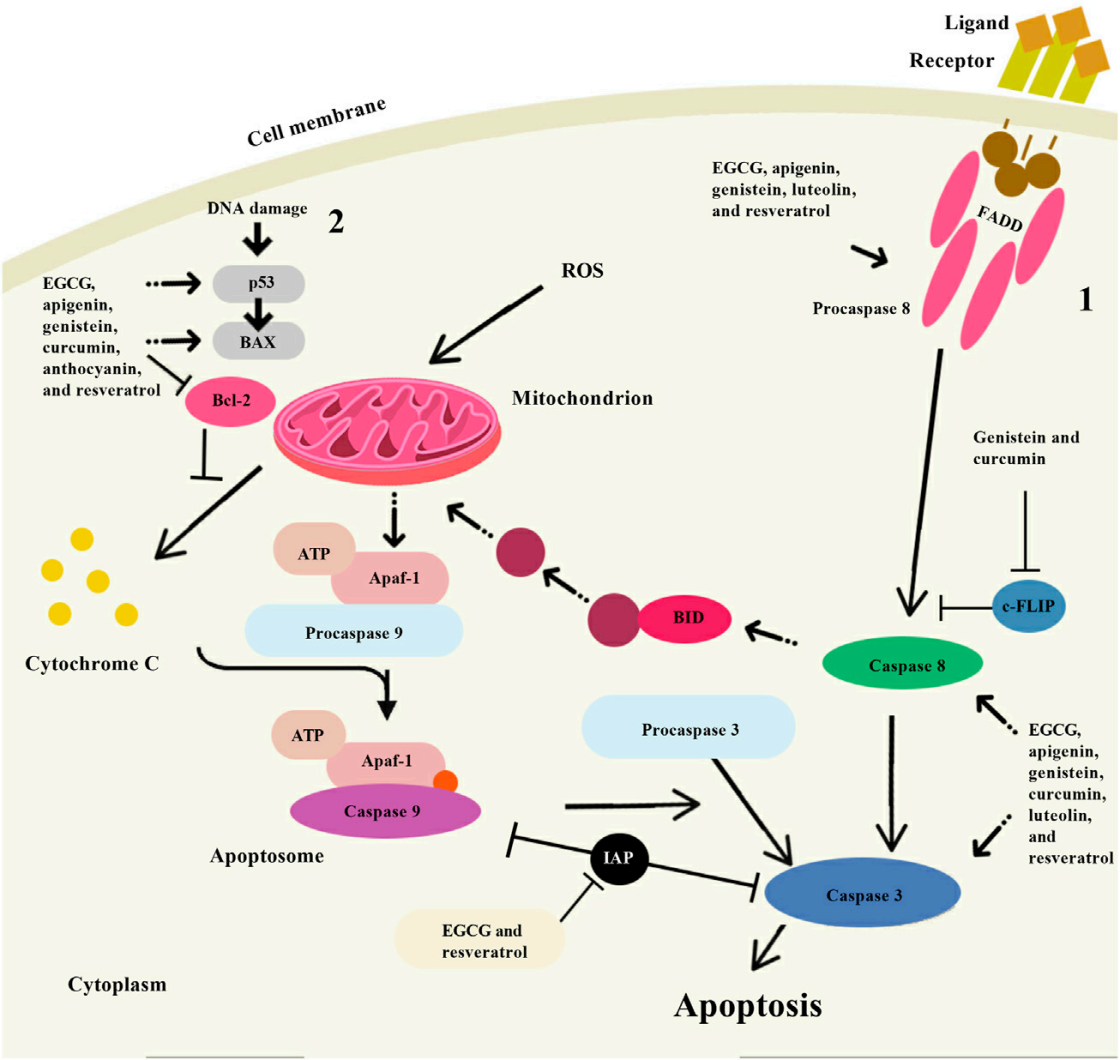
Apoptosis induction by some natural polyphenols. There are two main ways of apoptosis: 1. Apoptosis is activated by the interaction of specific ligands on the cell surface with receptor proteins containing “death domains”; and 2. The mitochondrial pathway of apoptosis begins with the collapse of the mitochondrial membrane potential and is accompanied by the release of cytochrome C from the mitochondrial intermembrane space into the cell cytoplasm. The vast experimental material obtained so far indicates that some natural polyphenols (epigallocatechin-3-gallate (EGCG), apigenin, genistein, luteolin, resveratrol, curcumin, and anthocyanin) have an apoptogenic effect using a variety of cellular targets. Note: BAX, Bcl-2 associated X-protein; Bcl-2, B-cell lymphoma-2; ROS, Reactive oxygen species; FADD, Fas-associated death domain; c-FLIP, Cellular FLICE-like inhibitory protein; Apaf-1, Apoptotic protease activating factor 1; ATP, Adenosine triphosphate; BID, BH3 interacting-domain death agonist.

Several mechanisms of action have been identified for the chemopreventive action of polyphenols, including estrogenic/antiestrogen activity, antiproliferative activity, apoptosis induction, cell cycle inhibition, oxidation prevention, detoxification enzyme induction, regulation of the host immune system, and anti-inflammatory activity (Singh et al., 2021a; Bahrami et al., 2021; Weh et al., 2022) (Figure 2, Figure 3 and Figure 4). At the same time, polyphenols can affect the metabolism of pro-carcinogens and modulate the expression of cytochrome P450 enzymes involved in their activation into carcinogens. They may also facilitate their clearance by increasing the expression of phase II conjugating enzymes. This induction of phase II enzymes may be due to polyphenol toxicity (Diaz-Gerevini et al., 2016).

Polyphenols can form potentially toxic quinones in the body, which are themselves substrates for these enzymes. Consumption of polyphenols can then activate these enzymes to detoxify themselves and thus cause an overall increase in the body’s defenses against toxic xenobiotics (Erlank et al., 2011). It has been demonstrated that tea catechins in the form of capsules, when administered to men with high grade intraepithelial neoplasia of the prostate, have a prophylactic activity by inhibiting the transformation of high grade intraepithelial neoplasia lesions into tumors (Bettuzzi et al., 2006).

Theaflavins, thearubigins and black tea polyphenols also had strong antitumor properties. Black tea polyphenols have been found to inhibit proliferation and enhance apoptosis in prostate carcinoma DU 145 cell lines (Klein and Fischer, 2002). Higher levels of insulin like growth factor-1 (IGF-1) have been found to be associated with a higher risk of developing prostate cancer (Cao et al., 2015). The binding of IGF-1 to its receptor is part of the signal transduction pathway that causes cell proliferation. The addition of black tea polyphenols was found to block the progression of IGF-1-induced cell growth into the S-phase of the cell cycle at a dose of 40 mg/ml in prostate cancer.

Polyphenols act as generators of reactive oxygen species (ROS) that act as second messengers in cellular signal transduction. In tumor cells, there are many targets that polyphenolic compounds can act on. However, nuclear factor kappa B (NF-κB) can be considered a central target, since it controls the expression of genes responsible for tumor proliferation, apoptosis, and metastasis (Khan et al., 2020).

Resveratrol prevents all stages of tumor development and has been shown to be effective in most types of tumors, including lung cancer, melanoma, breast cancer, prostate cancer, gastric cancer, and colorectal cancer (Rauf et al., 2018). It has also been shown to inhibit angiogenesis and metastasis. Extensive data *in vitro* show that resveratrol can modulate multiple signaling pathways involved in cell growth, apoptosis, and inflammation (Wu et al., 2019). The anticarcinogenic effects of resveratrol appear to be closely related to its antioxidant activity, where it inhibits cyclooxygenase, hydroperoxidase, protein kinase C, B-cell lymphoma 2 (Bcl-2) phosphorylation, Akt-kinase with focal adhesion, NF-κB expression, expression of matrix metalloproteases (MMPs) and cell cycle regulators (Yousef et al., 2017; El-Readi et al., 2019; Buhrmann et al., 2020). These and other *in vitro* and *in vivo* studies justify the use of polyphenols in tumor chemoprophylaxis in a combinatorial approach with chemotherapeutic drugs or cytotoxic factors for the effective treatment of drug-resistant tumors (Patra et al., 2021).

Based on the literature data on the effects of polyphenolic compounds on tumors, it can be concluded that polyphenols have great potential for use in combination with chemotherapy. A suitable combination of polyphenols with existing chemotherapeutic agents will result in a reduction in side effects without reducing the therapeutic effects of chemotherapy drugs.

### 2.1 Curcumin

Curcumin is the main curcuminoid found in turmeric root. Curcuminoids also include dimethoxycurcumin and bisdimethoxycurcumin. Turmeric has been used for its medicinal properties for thousands of years and is a widely used spice in Asian and Indian foods. Curcumin has a characteristic yellow color, is poorly soluble in water, but readily soluble in alcohol and dimethyl sulfoxide (DMSO). Curcumin has many health benefits, such as relieving inflammation, pain, and symptoms of metabolic syndromes. There is also evidence that curcumin has anti-tumor properties (Gupta et al., 2013; Lestari and Indrayanto, 2014).

Curcumin has been researched to have anticancer properties, but most of these studies have been done *in vitro*. These studies suggest that curcumin inhibits proliferation, induces cell cycle arrest and apoptosis through different mechanisms, in different types of tumor cell lines. There is evidence that curcumin reduces the expression of many different enzymes, transcription factors, inflammatory cytokines, growth factors, and other cell signaling components that are important for tumor growth and progression (Table 2) (Lee et al., 2011; Wang et al., 2019a; Zhang et al., 2020; Ebrahimi et al., 2021; Liang et al., 2021; Mao et al., 2021; Shi et al., 2021; Sun and Fang, 2021). For instance, in many studies of various tumor cell lines, curcumin has been found to downregulate the expression of the transcription factor NF-κB, which is normally highly expressed in tumor cells and is known to promote development, metastasis and tumor growth. In addition, curcumin stops the cell cycle in the G1/S or G2/M phases by inhibiting various cyclins in tumor cells (Figure 2). Curcumin also induces tumor cell apoptosis through caspase-dependent pathways and reduces the expression of anti-apoptotic proteins. Moreover, to *in vitro* studies, the properties of curcumin have also been studied in *in vivo*; overall demonstrating that curcumin has anti-proliferative effects (Lee et al., 2011; Wang et al., 2019a; Zhang et al., 2020; Ebrahimi et al., 2021; Liang et al., 2021; Mao et al., 2021; Shi et al., 2021; Sun and Fang, 2021). For instance, a mouse model of colorectal cancer treated with an intraperitoneal injection of curcumin demonstrated that curcumin inhibits tumor growth with favorable prognosis for overall survival. These data also suggest that curcumin activated miR-130a, which reduced expression of the Wnt/β-catenin signaling pathway and resulted in increased overall survival (Dou et al., 2017).

**TABLE 2:** Results of some preclinical experiments on the study of the therapeutic effect of curcumin in tumors.

Tumor type	Study model	Gene-targets	Mechanism of action	References
Breast cancer	*In vitro* and *in vivo*	ROS/YAP1/JNK signaling pathway	Suppresses tumor growth and metastasis. Induces tumor cell apoptosis	Wang et al. 2019a)
Gastric cancer	*In vitro*	Gli1-β-catenin	Decreases cellular migration and invasion, while enhances tumor cells apoptosis. Induces cytoskeletal remodeling and inhibits the epithelial-mesenchymal transition process	Zhang et al. (2020)
Colorectal cancer	*In vitro*	TFAP2A-mediated ECM pathway: GP1BB, COL9A3, COMP, AGRN, ITGB4, LAMA5, COL2A1, ITGB6, ITGA1, and TNC	Inhibits tumorsphere formation, decreases cell viability in a dose-dependent manner, and promote apoptosis	Mao et al. (2021)
Colorectal cancer	*In vitro*	p53, p21, BAX, BCL-2, and NOXA	Suppresses the proliferation of cancer cells via induction of apoptosis	Ebrahimi et al. (2021)
Ovarian cancer	*In vitro* and *in vivo*	circ-PLEKHM3/miR-320a/SMG1 axis	Suppresses cancer cell proliferation and promote apoptosis	Sun and Fang, (2021)
Papillary thyroid cancer	*In vitro*	MMP-2, MMP-9, miR-301a-3p/JAK/STAT3 axis	Inhibits the viability, migration and invasion	Liang et al. (2021)
Glioblastoma	*In vitro*	AKT pathway, Bcl-2	Anti-proliferation effect, suppresses the growth of tumor and reduces of apoptosis	Shi et al. (2021)
Medulloblastoma	*In vitro* and *in vivo*	HDAC	Induce apoptosis and cell cycle arrest at the G2/M phase. Reduces tumor growth and significantly increases mouse survival	Lee et al. (2011)

ROS, reactive oxygen species; YAP1, yes-associated protein 1; JNK, c-Jun N-terminal kinases; Gli1, GLI family zinc finger 1; TFAP2A, transcription factor AP-2 alpha; ECM, extracellular matrix; GP1BB, glycoprotein Ib (platelet), beta polypeptide; COL9A3, collagen type IX alpha 3 chain; COMP, cartilage oligomeric matrix protein; AGRN, agrin; ITGB4, integrin subunit beta 4; LAMA5, laminin subunit alpha-5; COL2A1, collagen type II alpha 1 chain; ITGB6, integrin subunit beta 6; ITGA1, integrin alpha-1; TNC, tenascin C; BAX, Bcl-2 associated X-protein; BCL-2, B-cell lymphoma-2; NOXA, phorbol-12-myristate-13-acetate-induced protein 1; MMP-2, matrix metalloproteinase-2; MMP-9, matrix metalloproteinase-9; JAK, janus kinase 2; STAT3, signal transducer and activator of transcription 3; HDAC, histone deacetylase 1.

Curcumin also downregulates NF-κB expression in an animal model of breast cancer, with subsequent inhibition of metastasis and angiogenesis. Studies in an animal model of pancreatic cancer have also shown that curcumin inhibits tumor growth, suppresses proliferation, and inhibits angiogenesis (Bimonte et al., 2016; Coker-Gurkan et al., 2018). These studies on the anticancer effects of curcumin are mostly pilot studies; however, curcumin has been shown to be non-toxic and well tolerated even at high concentrations. A Phase 2 pilot study evaluated the use of curcumin with docetaxel in 26 men with metastatic castration-resistant prostate cancer (mCRPC) (Passildas-Jahanmohan et al., 2021). In this study, the men received 6 g of curcumin daily in addition to docetaxel and prednisone for 6 cycles. There was a prostate-specific antigen (PSA) response among 59% of patients, and 40% experienced a partial response. There were no side effects in 100% of patients. While there appear to be countless therapeutic benefits with curcumin supplementation, most of these benefits are due to its antioxidant and anti-inflammatory effects.

One of the main problems of curcumin is its low bioavailability, which seems to be mainly due to poor absorption, rapid metabolism and rapid elimination. Polyphenols have been tested that will improve the bioavailability of curcumin. Most of them were designed to block the metabolic pathway of curcumin in order to increase its bioavailability (Anand et al., 2007). For instance, piperine, a well-known bioavailability enhancer, is the main active ingredient in black pepper and has been associated with a 2000% increase in the bioavailability of curcumin (Haq et al., 2021). Preclinical and clinical evidence suggests that curcumin has antitumor properties, and these data warrant its further evaluation as a potential treatment for tumors in humans. However, due to its poor bioavailability, ongoing research is developing approaches to improve its delivery to cells, thereby potentially improving dosage and efficacy. Thus, the problem of poor bioavailability seems to be eliminated by the addition of agents such as piperine, which increase bioavailability, thereby creating a curcumin complex.

Curcumin also shows significant interactions with cardiolipin, a lipid found exclusively in the mitochondrial membrane. It has been studied that curcumin influences the structure and dynamics of cardiolipin-containing biomimetic and biological membranes of mitochondria. The application of several biophysical methods reveals cardiolipin-stimulated association and internalization of curcumin into the lipid bilayer. In parallel, curcumin with cardiolipin-containing bilayers increased their fluidity and reduced lipid order (Taylor et al., 2011). These data suggest that membrane modifications mediated by cardiolipin interactions may play a role in the therapeutic functions of curcumin and that the mitochondrial inner membrane as a whole may represent a potential therapeutic target.

### 2.2 Carnosic acid

One of the polyphenolic compounds of plant origin that attracts the attention of clinicians is carnosic acid. The body is not able to synthesize carnosic acid on its own, so it can only get through food. Carnosic acid is a chemical compound that is found in large quantities in rosemary and sage, has a number of properties that make it possible to include it in some medicines and prophylactic products (Birtić et al., 2015). Rosemary leaves are used as a seasoning and food preservative. These fragrant herbs are not only valued for their unique taste and smell, but also chemical composition of spices has served as an excellent raw material for obtaining a useful medicinal compound - carnosic acid (Moore et al., 2016). Rosemary leaves are included in the British Herbal Pharmacopoeia (BHP). In the United States, India, China, they are the official pharmacological raw materials and are used in herbal medicine and homeopathy. Rosemary leaves are used as a seasoning and food preservative. Rosemary essential oil exhibits pronounced antibacterial, antifungal, anti-inflammatory, cytostatic, and antioxidant properties (Bahri et al., 2016).

In preclinical studies, it was proved that сarnosic acid had anti-inflammatory, antioxidant and antitumor activity (Bahri et al., 2016; Donmez et al., 2020; Hosokawa et al., 2020). The main property of this compound is that сarnosic acid has a high ability to neutralize free radicals, even in the human brain, preventing disorders associated with weakened nerve signal transmission. It is well known that impaired neural signaling, such as low neurotransmitter levels, is associated with mental illness, increased sensitivity to stress, and cognitive impairment. The use of сarnosic acid is possible for the prevention or treatment of conditions associated with reduced transmitter/mediator activity of dopamine, serotonin and norepinephrine (de Oliveira, 2018). ROS can cause inflammation, the development of cancer and simply lead to aging (Brieger et al., 2012). Tumors such as prostate cancer, colorectal cancer and breast cancer acquire metabolically highly active mitochondria with an increased frequency of respiratory complexes and a higher level of mitochondrial membrane potential and calcium retention (Brieger et al., 2012). Therefore, in such oxidative tumor types, the treatment strategy should be aimed at disabling oxidative mitochondrial phosphorylation. It has been investigated that the growth arrest caused by this polyphenol does not limit its protonophoric activity, but also depends on the alteration of mitochondria containing proteins that regulate the cell cycle. Targeting tumor metabolism with carnosic acid represents a promising strategy to overcome drug resistance and tumor sensitization in cancer therapy. Table 3 presents a number of preclinical studies showing the antitumor activity of the сarnosic acid (de Souza et al., 2021; de Oliveira et al., 2019; de Oliveira et al., 2016; Coyne and Narayanan, 2019; Corveloni et al., 2020; Liu et al., 2018a; D'Alesio et al., 2017; Min et al., 2021; Shao et al., 2022; Hasei et al., 2021; Liu et al., 2018b; Park et al., 2014; Petiwala et al., 2016; Kim et al., 2016).

**TABLE 3:** Results of some preclinical experiments on the study of the therapeutic effect of carnosic acid in tumors.

Tumor type	Study model	Gene-targets	Mechanism of action	References
Neuroblastoma	*In vitro*	PI3K/Akt/Nrf2/γ-GCL/GSH axis	Promotes mitochondrial protection	de Souza et al. (2021)
	*In vitro*	Nrf2	Promotes mitochondrial protection and pro-apoptotic stimulus	de Oliveira et al. (2019)
	*In vitro*	PI3K/Akt/Nrf2 axis	Promotes mitochondrial protection, suppresses mitochondria-related pro-oxidant and mitochondria-dependent pro-apoptotic effects of chlorpyrifos	de Oliveira et al. (2016)
Pulmonary adenocarcinoma	*In vitro*	Dexamethasone-(C21- phosphoramidate)-[anti-EGFR]	Anti-neoplastic cytotoxicity	Coyne and Narayanan, (2019)
Lung cancer	*In vitro*	PUMA	Cytotoxic activity, cell cycle arrest at G0/G1 and G2/M phases, and anti-apoptotic, effects	Corveloni et al. (2020)
	*In vivo*	iNOS2, Arg-1, and MMP-9	Anti-growth and pro-apoptotic effects	Liu et al. (2018a)
Breast cancer	*In vitro*	PI3K/AKT/mTOR, CDKN1A/p21WAF1 and CDKN1B/p27KIP1 signaling pathways	Late autophagy and causes derangement of the lysosomal compartment	D'Alesio et al. (2017)
Oral squamous cell carcinoma	*In vivo*	Bax, Bad, Caspase-3 and -9, PARP1, Bcl-2	Inhibits the tumor growth without affecting the body weight and tissue morphology	Min et al. (2021)
Glioma	*In vivo*	Cyclin B1, PARP, caspase-3, p-AKT, p62, LC3-I, and LC3-II	Enhances TMZ-induced inhibition of colony formation and cell migration and enhances TMZ-induced cell cycle arrest and cellular apoptosis	Shao et al. (2022)
Hepatocellular carcinoma	*In vitro*	AMPK	Suppresses cell proliferation and reduce cell viability	Hasei et al. (2021)
Chronic myeloid leukemia	*In vitro*	miR-780	Induction of apoptosis and cell cycle arrest	Liu et al. (2018b)
Melanoma	*In vitro*	Src, FAK, and AKT	Suppresses the adhesion of tumor cells, as well as the secretion of MMP-9, TIMP-1, uPA, and VCAM-1. Inhibits of the epithelial-mesenchymal transition	Park et al. (2014)
Prostate cancer	*In vitro* and *In vivo*	PERK, ATF-6 and IRE1α	Inhibits tumor growth	Petiwala et al. (2016)
Colon cancer	*In vitro*	p53, Bax, Mdm2, Bcl-2, and Bcl-xl, caspase-9, and -3, PARP, JAK2, Src kinases, STAT3, cyclin D1, D2, and D3	Induces apoptosis, inhibits the constitutive phosphorylation, inhibits cell viability and the expression of cyclin D1 and surviving	Kim et al. (2016)

PI3K, phosphoinositide 3-kinases; γ-GCL, γ-glutamate—cysteine ligase; GSH, glutathione; Nrf2, nuclear factor erythroid 2-related factor 2; EGFR, epidermal growth factor receptor; PUMA, p53 upregulated modulator of apoptosis; iNOS2, nitric oxide synthase 2; Arg-1, arginase 1; MMP-9, matrix metallopeptidase 9; mTOR, mammalian target of rapamycin; CDKN1A, cyclin-dependent kinase inhibitor 1A; CDKN1B, cyclin-dependent kinase inhibitor 1B; p27KIP1, p27; Bax, Bcl-2 associated X-protein; Bad, BCL2-associated agonist of cell death; PARP1, Poly [ADP-ribose] polymerase 1; Bcl-2, B-cell lymphoma-2; PARP, poly(ADP-Ribose) polymerase 1; p-AKT, phospho-Akt; LC3-I, microtubule-associated protein 1A/1B-light chain 3-I; LC3-II, microtubule-associated protein 1A/1B-light chain 3-II; AMPK, AMP-activated protein kinase; Src, proto-oncogene tyrosine-protein kinase Src; FAK, focal adhesion kinase; PERK, protein kinase R (PKR)-like endoplasmic reticulum kinase; ATF-6 a, activating transcription factor 6; Mdm2, mouse double minute 2; Bcl-xl, B-cell lymphoma-extra large; JAK2, janus kinase 2; TMZ, temozolomide; TIMP-1, tissue inhibitor of metalloproteinase 1; uPA, urokinase plasminogen activator; VCAM-1, vascular cell adhesion molecule 1.

## 3 Mechanisms of action of flavonoids

One of the promising sources of phytopreparations are medicinal plants containing flavonoids, which, due to their wide distribution in plants and great structural diversity, are currently in the focus of attention of researchers in the field of pharmacognosy, pharmacy and medicine. Flavonoids are the most numerous class of natural phenolic compounds, which are characterized by structural diversity, high and versatile activity and low toxicity. The wide range of biological activity of flavonoids is associated with the diversity of their chemical structures and the various physicochemical properties resulting from them (Figure 1). This interest is due to the fact that flavonoids cause antioxidant, angioprotective, hepatoprotective, and antitumor activity (Serafini et al., 2010; Wen et al., 2021). Moreover, it is the antitumor properties that attract scientists to the greatest extent in the field of creating new drugs in the fight against tumor.

Cell signaling systems are involved in the transmission of chemical signals from the cell surface to the cytoplasm, due to which the cell is able to respond to environmental changes. To do this, cells have specialized receptors on the surface of the plasma membrane that can recognize the presence of certain molecules in the environment, called extracellular signaling molecules. Flavonoids are able to influence the functioning of cytokine receptors, tyrosine kinase receptors (RTKs), tumor necrosis factor ligand superfamily member 10 (TRAIL), protein-coupled receptors (GRCRs), and a broad class of transmembrane signaling protein called integrins (Wiseman et al., 2001; Chen et al., 2018). The molecular mechanisms of this influence and signal propagation pathways are not well understood. In recent years, only scattered information has appeared indicating changes in the activity or expression of proteins of a particular signaling system in the presence of certain flavonoids. In this chapter, we will discuss the main mechanisms of action of flavonoids on the signaling pathways of oncogenesis.

### 3.1 Cytokine receptors

It is now known that polyphenolic compounds can affect the functioning of receptors for cytokines such as tumor necrosis factor alpha (TNF-α) or some interleukin receptors, which can be used in tumor therapy. To a large extent, therapy is provided by suppressing the production of inflammatory cytokines, which leads to a decrease in the binding of the nuclear factor NF-kB to DNA and a decrease in the production of a number of cytokines such as interleukin-1β (IL-1β), interleukin-6 (IL-6), interleukin-8 (IL-8) and TNF-α (Leyva-López et al., 2016).

Epigallocatechin-3-gallate (EGCG) is one of the most active green tea flavonoids, has the ability to normalize many cellular processes by neutralizing the damaging effects of high concentrations of cytokines that occur during inflammation (Ohishi et al., 2016). Thus, when acting on insulin-producing pancreatic β-cells, EGCG protected against the action of IL-1β and TNF-α and restored the ability of cells to produce insulin under the influence of glucose (Zhang et al., 2011). At the same time, the content of oxidation products and ROS in the cytoplasm decreased, the potential on the mitochondrial membranes was restored, the release of cytochrome c from mitochondria into the cytoplasm stopped, and the concentration of nitrogen oxide in the cytoplasm decreased due to the suppression of the expression of nitric oxide synthase genes under the action of cytokines (Jeong and Kim, 2021). Which suggests prophylactic properties to prevent the PC development. Hoffman et al. demonstrated that EGCG was able to block constitutive and IL-1-dependent NF-kB activation as well as production of tumorigenic factors in pancreatic cancer (Hoffmann et al., 2011). EGCG inhibited tumor growth, invasion, and metastasis implying an association of the EGCG-mediated downregulation of IL-1 with the reduction in tumor growth and the development of a malignant phenotype that might play a crucial role in oncogenesis of pancreatic cancer. In other study, was demonstrated that EGCG inhibited IL-6-induced vascular endothelial growth factor (VEGF) expression in gastric cancer cells, and this inhibitory effect was at transcriptional level (Zhu et al., 2011). The preclinical studies using flavonoids in tumors therapy through control the expression inflammation mediators are shown in Table 4 (Nicholas et al., 2007; Cai et al., 2011; Ma et al., 2011; Khan et al., 2012; García-Zepeda et al., 2013; Youn et al., 2013; Abaza et al., 2015; Zhang et al., 2015; Erdogan et al., 2016; Zhao et al., 2016; Zhang et al., 2019).

**TABLE 4:** The antitumor effects of flavonoids through control the expression inflammation mediators.

Flavonoids	Tumor type	Study model	Gene-targets	Mechanism of action	References
Quercetin	Lung cancer	*In vitro*	NF-κB	Induce tumor cell apoptosis	Youn et al. (2013)
Luteolin	Lung cancer	*In vitro*	NF-kB (p65)	Induce TNF-mediated apoptotic cell death	Cai et al. (2011)
EGCG	Lung cancer	*In vitro*	AP-1, MAPK, NF-κB, and COX-2	Inhibits tumor growth and metastasis	Zhang et al. (2019)
Genistein	Gastric cancer	*In vitro* and *In vivo*	NF-κB/COX-2	Inhibits angiogenesis and metastasis. Suppresses mortality, tumor number, tumor burden and chemical-induced inflammatory responses	(Ma et al., 2011), (Khan et al., 2012)
Quercetin	Colorectal cancer	*In vitro*	NF-κB	Induce tumor cell apoptosis	Zhang et al. (2015)
Naringenin	Colorectal cancer	*In vitro*	NF-κB/p65	Induce apoptosis and cell cycle arrest	Abaza et al. (2015)
Xanthohumol	Liver cancer	*In vitro*	NF-κB/p53	Induce apoptosis, modulating the NF-κB/p53 and the Notch1 signaling pathways	Zhao et al. (2016)
Xanthohumol	Cervical cancer	*In vitro*	NF-κB	Decrease expression of CXCR4, inhibits cell invasion induced by CXCL12	García-Zepeda et al. (2013)
Apigenin	Prostate cancer	*In vitro*	NF-κB/Akt	Induce apoptosis, inhibits cell invasion, motility	Erdogan et al. (2016)
Apigenin	Breast cancer	*In vitro*	NF-κB	Reduce TNF-α and IL-1β expression	Nicholas et al. (2007)

EGCG, epigallocatechin-3-gallate; IL-1β, interleukin -1β; NF-κB, nuclear and cytoplasmic nuclear factor kappa B; TNF-α, tumor necrosis factor-alpha; MAPK, mitogen-activated protein kinase; COX-2, cyclooxygenase 2; AP-1, activating protein-1; CXCR4, C-X-C chemokine receptor type 4; CXCL12, chemokine (C-X-C motif) ligand 12.

### 3.2 TRAIL-induced apoptosis pathway

The role of TRAIL has not been sufficiently studied, but it has been shown that this protein plays a role in the formation of T-lymphocyte memory, in the processes of hematopoiesis, in the development of autoimmune diseases, and in many other phenomena (Falschlehner et al., 2009). TRAIL plays a significant role in the antitumor activity of T-lymphocytes and natural killer cells (NK cells) (Falschlehner et al., 2009). Thus, TRAIL regulates the growth and metastasis of tumors, which is an important part of the body’s immune defense against the development of oncogenesis (Cardoso Alves et al., 2021). This protein contains 281 amino acids and is a homotrimer that combines three identical molecules. TRAIL is found on the surface of some immune cells (T cells, NK cells) (Falschlehner et al., 2009). There is also a water-soluble form of the TRAIL protein. The soluble form of TRAIL exhibits less liver toxicity than the membrane-bound form and can be used to initiate tumor cell apoptosis. The TRAIL molecule circulating in the blood binds to the transmembrane cell death receptors DR4 (TRAIL-R1) or DR5 (TRAIL-R2) located on the plasma membrane of cancer cells, which triggers a cascade of chemical processes leading to apoptosis (Yuan et al., 2018). The apoptosis factor TRAIL is synthesized by immune cells (T- and NK-cells), attaches to the DR4/DR5 cell death receptors on the surface of tumor cells, after which the death-inducing signaling complex (DISC) is formed, which also involves the adapter FAS-associating death domain-containing protein (FADD) and procaspase-8 or -10. Subsequently formed caspase-8 or -10 activates caspase-3 (possibly also -6 or -7), which is an effector of apoptosis (Voss et al., 2021). This path is called external. It can be influenced by the apoptosis regulator FADD-like IL-1β-converting enzyme)-inhibitory protein (c-FLIP) protein (also called caspase 8). It is also possible to activate caspase-3 through mitochondria (MTX). In this case, caspase-8 or -10 activates the apoptosis agonist BH3 interacting-domain death agonist (BID) protein (another designation BH3), which acts on mitochondrial membranes through Bax and/or Bak proteins, resulting in the formation of pores in the outer mitochondrial membrane, through which cytochrome c (Cyt C) (Engels et al., 2005; Crowder and El-Deiry, 2012). The latter through caspases is able to initiate apoptosis. The action of the Bax protein is regulated by its associated apoptosis regulatory proteins Bcl-2, BCL-xL and the inducible myeloid leukemia cell differentiation protein induced myeloid leukemia cell differentiation protein (Mcl-1). The action of caspase-9 and caspase-3 can be modulated by the apoptosis inhibitors X-linked inhibitor of apoptosis protein (XIAP), calf intestinal alkaline phosphatase (CIAP) and Survivin, which are regulated by the mitochondrial caspase activator SMAC (also known as Diablo) (Singh et al., 2021b). Mitochondrial damage can also be caused by the tumor suppressor protein p53, which acts in the presence of ROS or Akt protein kinase, which in turn is activated by phosphoinositide 3-kinase (PI3K) (Wendt et al., 2005). For unknown reasons, activation of the TRAIL signaling pathway does not cause toxicity to normal cells, which distinguishes this factor from TNF-a or FasL (Falschlehner et al., 2009). The latter can also trigger apoptosis processes, but their use in medicine is very problematic, since these proteins are highly toxic to healthy cells of various organs, especially to hepatocytes.

Clinical trials using recombinant human TRAIL in combination with conventional chemotherapy have shown encouraging results. However, some tumor cells are resistant to activation of the TRAIL signaling pathway. Overcoming this resistance and increasing the ability of cells to apoptosis can significantly help in the treatment of various types of tumors (Deng and Shah, 2020; Thapa et al., 2020). Many polyphenolic compounds, in most cases flavonoids, show synergistic effects with TRAIL, affecting various proteins involved in the regulation of apoptosis, survival, or the rate of tumor cell division. Thus, Nishikawa et al. was the first to discover that green tea EGCG was able to enhance the effect of TRAIL on human hepatocarcinoma cells, through a negative regulatory effect on Bcl-2α and BCL-xL proteins (Nishikawa et al., 2007). A similar mechanism of action through the proteins Bcl-2, BCL-xL and a number of other proteins was found in the action of EGCG to TRAIL in prostate carcinoma cells (Thapa et al., 2020). Later, the effectiveness of kaempferol against TRAIL in relation to glioblastomas was shown, where the specified flavonoid initiated the degradation of survivin and inhibition of the Akt pathway, which led to the death of tumor cells (Yoshida et al., 2008). Quercetin may enhance the action of TRAIL through dephosphorylation of the Akt pathway and activation of caspases in human adenocarcinoma cells (Moon et al., 2015). However, no cytotoxicity was found in relation to normal cells. The same authors showed that quercetin is able to activate caspases-3, -8 and -9. The ability of quercetin to interact with the survivin promoter and prevent the expression of this protein was also found (Özsoy et al., 2020). Information on the effect of flavonoids on various components of the signaling system of TRAIL-dependent apoptosis is given in Table 5 (Ismail et al., 2015; Han et al., 2016; Park et al., 2016; Li et al., 2017; Xu et al., 2018; Kim et al., 2020; Wu et al., 2020; Huang et al., 2021).

**TABLE 5:** The antitumor effects of flavonoids by regulation the expression of TRAIL-induced apoptosis.

Flavonoids	Tumor type	Study model	Gene-targets	Mechanism of action	References
Irigenin	Gastric cancer	*In vitro* and *in vivo*	Caspase-8/-9/-3, PARP, FADD, DR5, Bax, c-FLIP, Bcl-2 and Survivin	Reduce tumor growth and suppresses tumor progression	Xu et al. (2018)
2-(3-hydroxyphenyl)-5-methylnaphthyridin-4-one (CSC-3436)	Triple-negative breast cancer	*In vitro*	c-FLIPS/L, Bcl-Xl, Bcl-2, Survivin, XIAP, and ROS/p38/C/EBP- CHOP signaling pathway	Sensitizes of tumor cell to chemotherapy	Huang et al. (2021)
Icariin	Colon cancer	*In vitro* and *In vivo*	ROS, ERK and CHOP	Sensitizes of tumor cell to chemotherapy and reduce tumor growth	Kim et al. (2020)
Luteolin	non-small cell lung cancer	*In vitro*	DR5 and Drp1	Sensitizes of tumor cell to chemotherapy	Wu et al. (2020)
2'-hydroxy-4-methylsulfonylchalcone (RG003)	Prostate cancer	*In vitro*	Bcl-2, PI3K/Akt, NF-κB, and COX-2	Increases poly-ADP-ribose polymerase cleavage and DNA fragmentation. Reduces inflammation and stimulate of apoptosis	Ismail et al. (2015)
Morusin	Glioblastoma	*In vitro*	EGFR, DR5, Survivin, XIAP, PDFGR, STAT3	Decreases cell viability and increases apoptosis	Park et al. (2016)
EGCG	Nasopharyngeal carcinoma	*In vitro*	Bcl-XL, Bcl-2, FADD, c-FLIP, caspase-8/-9/-3, p65, NF-κB, XIAP and Survivin	Modulate of intrinsic and extrinsic apoptotic pathways	Li et al. (2017)
Galangin	Renal carcinoma	*In vitro*	NF-κB, Bcl-2, c-FLIP, Mcl-1 and Survivin	Increases apoptosis and tumor growth	Han et al. (2016)

EGCG, epigallocatechin-3-gallate; PARP, poly (ADP-ribose) polymerase; FADD, FAS-associated protein with death domain; DR5, death receptor 5; c-FLIP, cellular-FLICE inhibitory protein; Bax, Bcl-2-associated X protein; Bcl-2, B-cell lymphoma 2; Bcl-Xl, B-cell lymphoma-extra-large; XIAP, X-linked inhibitor of apoptosis protein; ROS, reactive oxygen species; CHOP, C/EBP-homologous protein; ERK, extracellular signal-regulated kinase; DR5, death receptor 5; Drp1, dynamin-related protein 1; PI3K, phosphoinositide 3-kinases; NF-κB, nuclear factor kappa-light-chain-enhancer of activated B cells; COX-2, cyclooxygenase-2; EGFR, epidermal growth factor receptor; PDFGR, platelet-derived growth factor receptor alpha; STAT3, signal transducer and activator of transcription 3; FADD, FAS-associated death domain protein; Mcl-1, myeloid-cell leukemia 1.

### 3.3 Receptor tyrosine kinases

RTKs play an essential role in the regulation of processes associated with cell proliferation or death. In addition, this receptor is a therapeutic target for many drugs used in the treatment of tumors (Trenker and Jura, 2020). The receptor is a transmembrane protein with which various growth factors, cell division and some hormones interact. Accordingly, about 20 different types of RTK are distinguished. These include the insulin receptor (IR), epidermal growth factor receptor (EGFR), the ephrin (Eph) receptor, a protein that regulates intracellular interactions and cell migration, and the angiopoietin receptor responsible for angiogenesis (Choura and Rebaï, 2011).

Catechins may have a therapeutic effect on many types of scrotum cells, as well as on the development and progression of tumors *in vivo*, due to the suppression of RTK signals (Larsen et al., 2010). Being located in the plasma membrane, this receptor is sensitive to changes in the physical properties of lipids, which can be influenced by flavonoids. Among them, catechins are perhaps one of the most effective anticarcinogenic agents among plant polyphenols. One possible explanation for their activity suggests that flavonoids are mimetics of the adenine portion of the adenosine triphosphate (ATP) molecule and are able to block the ATP-binding sites of protein kinase receptors (Baranwal et al., 2022). In addition, attention is drawn to their ability to influence the lateral segregation of plasma membrane lipids and the formation of lipid rafts, which disrupts the functioning of membrane receptors, such as RTK or the epithelial growth factor receptor EGFR (Choura and Rebaï, 2011; Talukdar et al., 2020). Thus, EGCG prevents the binding of the epithelial growth factor to the corresponding receptor and inhibits the functioning of other RTKs, which determines the anticarcinogenic effect of this flavonoid (Shimizu et al., 2011). The flavonoid silibinin probably also has a similar effect on RTK (Rho et al., 2010). Table 6 presents preclinical studies on the inhibitory effect of some flavonoids on RTK activity in various tumors (Peterson and Barnes, 1993; Lee et al., 2004; Shimizu et al., 2010; Kim et al., 2014; Suh et al., 2015; Lee et al., 2017; Namiki et al., 2020; Kim et al., 2021).

**TABLE 6:** The antitumor effects of flavonoids by regulation of receptor tyrosine kinases (RTKs) and integrins.

Flavonoids	Tumor type	Study model	Gene-targets	Mechanism of action	References
Luteolin	Non-small cell lung cancer	*In vitro*	Tyro3, Axl and MerTK	Anti-proliferative effect	Lee et al. (2017)
EGCG	Lung cancer	*In vitro*	Axl	Inhibits stemness and tumourigenicity	Namiki et al. (2020)
Apigenin	Non-small cell lung cancer	*In vitro*	Axl, p21 and XIAP	Anti-proliferative effect	Kim et al. (2014)
Quercetin and luteolin	Pancreatic cancer	*In vitro*	FAK, PTK, EGFR, and MMP	Suppresses of invasive potential and cell migration. Induce apoptosis	Lee et al. (2004)
Apigenin	Ovarian cancer	*In vitro*	IL-6, STAT3, Bcl-xl, and Axl	Inhibits of apoptosis and tumor cells proliferation	Suh et al. (2015)
Quercetin	Glioblastoma	*In vitro*	Axl, IL-6 and STAT3	Induces apoptosis	Kim et al. (2021)
Genistein and biochanin A	Prostate cancer	*In vitro*	EGF	Inhibits the tumor growth	Peterson and Barnes, (1993)
EGCG	Colorectal cancer	*In vitro*	VEGFR, HIF-1alpha, IGF-1/2, epidermal growth factor (EGF), and heregulin	Inhibits of angiogenesis	Shimizu et al. (2010)

EGCG, epigallocatechin-3-gallate; MerTK, myeloid-epithelial-reproductive tyrosine kinase; XIAP, X-linked inhibitor of apoptosis protein; FAK, focal adhesion kinase; PTK, protein tyrosine kinases; EGFR, epidermal Growth Factor Receptor; MMP, matrix metalloproteinases; IL-6, interleukin-6; STAT3, signal transducer and activator of transcription 3; Bcl-xl, B-cell lymphoma-extra large; EGF, epidermal growth factor; VEGFR, vascular endothelial growth factor; HIF-1alpha, hypoxia-inducible factor 1-alpha; IGF-1/2, insulin-like growth factor 1/2; EGF, epidermal growth factor.

### 3.4 Integrins

Integrins are surface cellular receptors that transmit signals to the cytoplasm about changes in the chemical composition of the matrix surrounding cells. Integrins are present on the cell surface of most multicellular organisms, and usually consist of two α and β subunits that form 24 different dimeric molecules. Each subunit has a transmembrane segment, extracellular and cytoplasmic domains (Desgrosellier and Cheresh, 2010). Integrins are of great importance in the regulation of intercellular interaction, cell adhesion and migration. Integrins are involved in various diseases, including tumor development and metastasis processes (Sonnenberg, 1993). Accordingly, integrins serve as targets for the therapeutic effects of various drugs in tumors. EGCG is able to influence the activity of monocytes due to a decrease in the expression of α5β3 integrins, which is essential in the regulation of tumor growth and metastasis (Sen and Chatterjee, 2011). EGCG can inhibit the migration and adhesion of B-lymphocytes, which are also involved in the development of the immune response by blocking the expression of CD11b integrin (Kawai et al., 2004). EGCG is also able to suppress the expression of the EGFR through its action on the α5β1 integrin, which is of great importance in the development of human carcinoma (Ye et al., 2017). There are also data on the effect of EGCG on the motility and migration of fibroblasts due to the suppression of the expression of the α2β1 integrin, which may be important in the antitumor activity of this catechin (Hung et al., 2005). Apigenin, present in many medicinal herbs (chamomile, adonis, lemon balm, etc.) can block p5 integrin in breast cancer cells (Imran et al., 2020). Kaempferol, a flavonoid from cumin, tea, viburnum, and others, suppresses TNF-α-induced β2 integrin expression of eosinophils, which prevents them from infiltrating the airway epithelium *in vivo*, suggesting preventive properties for lung cancer (Kuo et al., 2015). Glabridin, a licorice flavonoid, suppresses the expression of αnuβ3 integrin, which, along with the suppression of the activity of some other components of the signaling system (FAC/Src, Akt, and Ras homolog family member A (RhoA)), prevents migration, invasion, and angiogenesis of lung cancer cells (Tsai et al., 2011). Table 7 presents preclinical studies on the inhibitory effect of some flavonoids on integrins activity in various tumors (Pilorget et al., 2003; Dastpeyman et al., 2012; Ruan et al., 2012; Deep et al., 2014; Li et al., 2018; Min et al., 2018; Liu et al., 2021; Qiao et al., 2022).

**TABLE 7:** The antitumor effect of flavonoids by regulation of integrins.

Flavonoids	Tumor type	Study model	Gene-targets	Mechanism of action	References
Pristimerin	Triple-negative breast cancer	*In vitro* and *in vivo*	E-cadherin, N-cadherin and integrin β3	Inhibits tumor growth and EMT reversion	Liu et al. (2021)
Deguelin	Non-small cell lung cancer	*In vitro* and *in vivo*	CtsZ/FAK/Src/Paxillin and integrin β3	Anti-metastatic effect	Li et al. (2018)
Baicalein	Gastric cancer	*In vitro*	miR-7/FAK/AKT signaling pathway	Inhibits cell proliferation, metastasis and angiogenesis	Qiao et al. (2022)
Erybraedin A	Non-small-cell lung cancer	*In vitro*	Integrin β1, integrin β3 and Src	Block the Src-mediated adhesion and survival of tumor cells	Min et al. (2018)
Silibinin	Prostate cancer	*In vitro* and *in vivo*	Integrins (α5, αV, β1 and β3), FAK, Src, GTPases, ARP2 and cortactin, cPARP, caspase 3), E-cadherin, β-catenin, survivin, and Akt	Inhibits tumor cells motility, invasiveness and survival	Deep et al. (2014)
Luteolin	Non-small cell lung cancer	*In vitro*	Integrin β1 and FAK	Inhibits hypoxia-induced proliferation, motility and adhesion in the cells	Ruan et al. (2012)
Silibinin	Highly metastatic human breast cancer	*In vitro*	β1-integrin, Raf-1, Cdc42 and D4-GDI	Inhibits proliferation, migration and adhesion of tumor cells	Dastpeyman et al. (2012)
EGCG	Medulloblastoma	*In vitro*	α2-integrin, α3-integrin and β1-integrin	Inhibits cell invasion	Pilorget et al. (2003)

EGCG, epigallocatechin-3-gallate; CtsZ, cathepsin Z; FAK, focal adhesion kinase; ARP2, actin related protein 2; cPARP, cleaved poly-ADP ribose polymerase; Cdc42, cell division cycle 42; D4-GDI, D4-guanine diphosphate (GDP)–dissociation inhibitor (GDI).

## 4 Crossing the blood-brain barrier

In order to assess the potential of various polyphenolic compounds to have an effect on tumors of the central nervous system (CNS), it is first necessary to consider the ability of these substances to crossing the blood-brain barrier (BBB). The bioavailability of polyphenolic compounds for the CNS is very low. For example, direct administration of large amounts of EGCG into the stomach during the day made it possible to obtain very high concentrations of this substance in the blood plasma, but its concentration in the brain was 5–10% of the concentration in the blood *in vivo* (Ferri et al., 2015). Thus, in order to achieve therapeutic concentrations of EGCG in the brain, it was necessary to increase its concentration in the blood to excessively high values. The study of other flavonoids showed that quercetin penetrates poorly through the BBB, but after penetrating, it accumulates in such parts of the brain as the hippocampus, striatum, cerebellum, where its concentration can reach 1 mg per gram of brain tissue (Pervin et al., 2019). Kaempferol and isorhamnetin penetrate better, and the average concentration of these substances in the brain can reach several hundred nanograms per gram of healthy tissue (Rangel-Ordóñez et al., 2010). At present, doubts have arisen about the adequacy of estimates of the degree of penetration of polyphenolic compounds into the CNS, as well as the effectiveness of the action of low concentrations of these substances, since, despite the apparent low content of these substances in the tissues of the CNS, there is a lot of experimental evidence of their effective effect on behavioral reactions and cognitive functions of animals and humans. In addition, it was found that after penetration into the brain tissue, flavonoids can undergo significant modification. Thus, catechins are conjugated with glycosides and are present in the form of glucuronides, which also have the ability to protect cells from oxidative stress. Moreover, chemical modification of flavonoids and other plant polyphenols can be used to deliver these substances to the brain, where they can be highly active (Rudrapal et al., 2021; Zhou et al., 2022). Thus, it is proposed to use a fully acetylated form of EGCG as a drug precursor. It was shown that active EGCG is released in the cytoplasm of cells due to the action of intracellular esterases (Lambert et al., 2006). The use of flavonoids as building blocks for the creation of substances capable of penetrating the BBB and exhibiting drug activity is one of the most promising research strategies in the therapy of CNS tumors.

## 5 Mechanisms of protective action against ultraviolet radiation

Prolonged intense exposure to UV radiation on the surface of the skin leads to the development of oxidative stress, damage to DNA molecules and the development of inflammatory processes (Aphalo et al., 2015). Exposure to ultraviolet light can cause various skin diseases, not only premature aging, but also serious diseases such as melanoma and non-melanoma skin cancers (Watson et al., 2016). Most polyphenolic compounds of plant origin are able to absorb radiation in the UV range and, therefore, can act as a screen. Indeed, it has been experimentally shown that when extracts from plants are applied to the surface of the skin, the reaction of the skin to ultraviolet light irradiation is significantly reduced (Cavinato et al., 2017). However, the protective effect of these substances is not limited to shielding tissues from the action of the ultraviolet part of the spectrum. Protection is also carried out due to the action on the regulatory systems of the cell.

It was found that the appearance of ROS is associated with the activation of the factor NF-kB and the subsequent expression of NADPH oxidase and cyclooxygenase-2 (COX-2), the activity of which is the cause of the accumulation of ROS in keratinocytes (Wang et al., 2019b). The activity of cyclooxygenase in the cytoplasm and the accumulation of products of lipid peroxidation (LPO) are associated with an increase in the concentration of calcium cations in the cytoplasm during ultraviolet irradiation. Antioxidants do not appear to be able to protect the skin from the effects of UV radiation (Sourivong et al., 2007). Thus, it was experimentally shown that the antioxidant ionol was not effective in protecting cells exposed to UV radiation, while the blocker of COX-2 cyclooxygenase aspirin reduced the concentration of lipid peroxidation in keratinocytes (Ponomareva et al., 1985). Due to the fact that UV radiation induces the activity of COX-2 cyclooxygenase, the concentration of prostaglandins formed from arachidonic acid increases in skin cells (De Leo et al., 1984). As a result, inflammation processes develop, edema is observed, keratinocyte proliferation accelerates, epidermal hyperplasia accelerates, oxidation products accumulate, which leads to oxidative damage to DNA. Therefore, as a result of chronic irradiation, mutations accumulate, which leads to malignant degeneration of keratinocytes and the development of oncogenesis (Leonardi et al., 2018). On the contrary, the action of COX-2 inhibitors or agents that interfere with the expression of this enzyme can significantly prevent the carcinogenic degeneration of epidermal cells (Leonardi et al., 2018).

The ability of some flavonoids to suppress the expression of COX-2 may underlie the mechanisms of the protective action of these substances against the action of UV radiation, as was shown for baicalein and wogonin, hesperetin, magniferin and tangeritin (Fischer et al., 2018; Farjadmand et al., 2021). It is possible that this regulation is carried out through the mitogen-activated protein kinase (MAPK) regulatory pathway, as was shown for luteolin (Aziz et al., 2018). Using the methylated flavonol 5,7-dimethoxyflavone as an example, it was shown that not only COX, but also other components of the regulatory chain, such as peroxisome proliferator-activated receptor (PPAR), NF-kB, can be regulated, resulting in a decrease in the concentration of IL-6 and IL-8 (Walle and Walle, 2007). In addition, there is a decrease in the expression of MMPs, a decrease in the concentration of oxidative stress components, and suppression of the activity of inflammation components through the regulatory pathways NF-kB and MAPK (Walle and Walle, 2007). As a result, damage to the skin and the subsequent possible development of oncology associated with the action of UV radiation are prevented.

Flavonoids may also affect other regulatory systems. Silibinin has recently been found to prevent damage to the epidermis after UV radiation by activating the tumor suppressor protein p53, resulting in the activation of the GADD45α protein, which contributes to the protection of cells under conditions of stress and DNA damage (Rigby et al., 2017). Chrysin protects epidermal keratinocytes from damage by UV irradiation, primarily by restoring the expression of aquaporin 3 (AQP-3), which ensures the normalization of the osmotic and salt balance of the cell, disturbed by irradiation. In a study on the protective effects of eriodictyol on keratinocytes, this flavanoid was found to act through activation of the p38/MAPK/Akt signaling pathway (Wu et al., 2011). Blackberry anthocyanins protect keratinocytes from UV radiation by significantly increasing the expression of antioxidant enzymes such as catalase, mitochondrial superoxide dismutase and glutathione peroxidase, thus preventing the development of oxidative stress (Murapa et al., 2012). Grape procyanidins also prevent the production of reactive oxygen species in cells, but using a different mechanism - suppression of p38 (MAPK14) and c-Jun NH1-terminal kinase/c-Jun NH2-terminal kinase (JNK1/2) (MAPK8) expression (Matito et al., 2011). The soy isoflavone metabolite daidzein, 7,3',4'-trihydroxy-isoflavone, prevents the development of melanoma induced by UV irradiation and by acting on the ATP-binding sites of protein kinases Cot and mitogen-activated protein (MAP) kinase kinase 4 (MKK4) (Ravindranath et al., 2004). It is noteworthy that the original daidzein molecule is not able to interact with these proteins and does not exhibit anticarcinogenic activity under these conditions.

## 6 Polyphenols and viruses

According to current data, viruses are the etiological agents of about 15% of human tumors. These viruses include: human T-leukemia/lymphoma virus, human immunodeficiency virus (HIV), human papillomavirus (HPV), hepatitis B and C viruses, Epstein-Barr virus (EBV), and other (Cao and Li, 2018). It is important to note that some viruses are associated with tumors of only one localization, while others are associated with various malignant neoplasms, which is probably due to the tropism of viruses for certain types of cell systems. The virus-genetic theory of the occurrence of tumors, proposed back in the 40 s of the 20th century by Zilber, has received numerous confirmations over the years (Cao and Li, 2018; Haley et al., 2019). At present, it is clear that although viruses are not the only cause of tumors, they play a large role in the occurrence of malignant neoplasms (for instance, the presence of hepatitis B virus increases the risk of developing hepatocellular carcinoma) in both humans and animals. A characteristic feature of tumor diseases associated with viruses is a long latent period, years and even decades can pass from the moment of infection to the manifestation of the disease (Mui et al., 2019; Blackard and Sherman, 2021).

**FIGURE 5:**
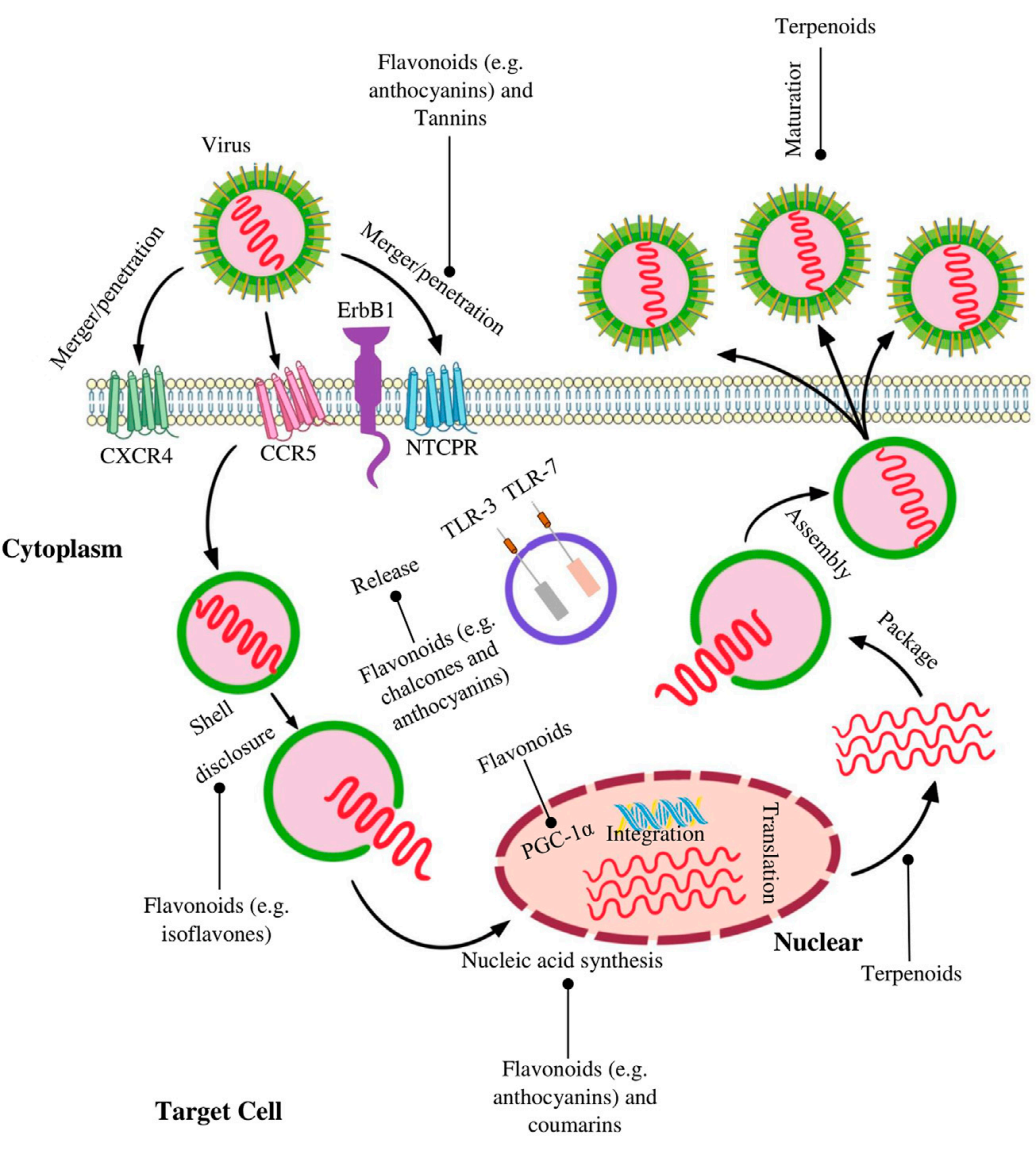
Targets and processes affected by some natural polyphenolic compounds in viral infections. This scheme shows the possible mechanisms of the antiviral action of the main classes of natural polyphenolic compounds. Flavonoids have been found to inhibit merger, integration, and reverse transcription. Inhibition of protease, reverse transcriptase, replication and maturation are among the anti-HIV mechanisms of some terpenoids. Coumarins inhibit transcriptase and activation of nuclear factor-kappa B (NF-kB). Note: PGC-1α, Peroxisome proliferator-activated receptor gamma coactivator 1-alpha; TLR-3, Toll-like receptor 3; TLR-7, Toll-like receptor 7; ErbB1, Receptor tyrosine-protein kinase; CCR5, C-C chemokine receptor type 5; CXCR4, C-X-C chemokine receptor type 4.

The creation of antiviral drugs based on natural compounds is undoubtedly one of the promising real directions. The expansion of the number of compounds used and the use of the synergism of their biological action provide a large reserve of therapeutic antiviral action and, along with the prevention of oncological diseases. Along with a number of different natural substances, polyphenolic compounds with antioxidant activity can also be used, in particular flavanoids, which occupies one of the leading positions in antioxidant activity. The mechanism of the antiviral action of polyphenolic compounds includes both direct interference with the mechanism of viral replication and suppression of cellular signaling pathways necessary for replication (Di Petrillo et al., 2022) (Figure 5). Numerous studies have shown that some polyphenols have an effect against influenza A, herpes virus, hepatitis B and C, HPV and others. Table 8 lists the polyphenols for which their antiviral activity has been confirmed (Docherty et al., 2004; Docherty et al., 2005; Yiu et al., 2010; Ciesek et al., 2011; Fatima et al., 2014; Pang et al., 2014; Rastogi et al., 2015; Sun et al., 2021).

**TABLE 8:** List of polyphenols with antiviral activity.

Polyphenols	Virus type	Targets/signaling pathways	Mechanism of action	References
Resveratrol	Human herpesvirus 1 and Human herpesvirus 2	ND	Significantly reduced skin lesions, and the effectiveness of the drug depended on its concentration, the time of initiation of treatment and the number of applications per day	(Docherty et al., 2004), (Docherty et al., 2005)
Epigallocatechin-3-gallate	Hepatitis C Virus	E1/E2	Inhibition of virion attachment	Ciesek et al. (2011)
Epigallocatechin-3-gallate	Hepatitis C Virus	GT3a and NS3 helicase	Binding interaction of virus NS3 helicase active pocket	Fatima et al. (2014)
Epigallocatechin-3-gallate	Hepatitis B Virus	HBsAg, HBeAg and virus DNA	Strong anti-HBV activity through decreasing the secretion of HBsAg, HBeAg and extracellular HBV DNA, although perhaps in such a way that the mechanism may interfere with the replication cycle of HBV DNA	Pang et al. (2014)
Resveratrol	Human papillomavirus	p-pRb1, p53, virus E6 and E7 genes	Inhibits cervical cancer development by suppressing the transcription and translation of E6 and E7, and also by promoting the apoptosis and G1/S phase transition arrest.	Sun et al. (2021)
6-Gingerol	Human papillomavirus	p53, p21, caspase-3 and PARP	Inhibits the chymotrypsin activity of proteasomes; induce reactivation of p53, and DNA damage and G2/M cell cycle arrest; increases levels of p21; and potentiates the cytotoxicity of cisplatin.	Rastogi et al. (2015)
Resveratrol	Epstein-Barr virus	Rta, Zta, and EA-D	Inhibits virus lytic cycle	Yiu et al. (2010)

GT3a, genotype 3a; NS3, nonstructural protein 3; HBsAg, hepatitis B surface antigen; HBeAg, hepatitis B e-antigen; p-pRb1, phosphorylated retinoblastoma protein; PARP, Poly (ADP-ribose) polymerase; HBV, Hepatitis B Virus; ND, not mentioned.

Significant progress has been made in the use of plant polyphenolic compounds for the treatment of HIV infection. Flavonoids have been found to inhibit viral fusion, integration, and reverse transcription. Inhibition of protease, reverse transcriptase, replication, and maturation are among the anti-HIV mechanisms of some polyphenols (Yu and Zhao, 2012; Andrae-Marobela et al., 2013). Flavonoids, alkaloids, anthocyanins, chalcones, xanthones and homoisoflavonoids, which inhibit neuraminidase, are proposed to be used as anti-influenza agents (Bahramsoltani et al., 2016). Polyphenol, isochlorogenic acid, dehydrocheilantifoline and some other amide alkaloids have an effect against hepatitis B virus (Man et al., 2021). Curcumin inhibits the replication and expression of the hepatitis B virus gene (Thongsri et al., 2021). In cell model studies, it was found that quercetin inhibits the entry of the influenza virus at an early stage of infection, due to its pronounced anti-inflammatory properties, reduces the effects of pro-inflammatory cytokines and the risk of developing lung inflammation (Mehrbod et al., 2020; Nile et al., 2020). In addition, it enhances the effect of other drugs.

In general, polyphenols are able to interfere with different stages of the life cycle of viruses, which characterizes them as multipurpose drugs that act on vital proteins of the pathogen (Table 8). At the same time, many researchers pay attention to the fact that for the development of drugs based on polyphenols, it is necessary to overcome quite a lot of difficulties, since these compounds are characterized by complex structures, low bioavailability, and rapid excretion from the body. In addition, in-depth studies *in vitro* (*ex vivo*), *in vivo*, as well as multicenter clinical studies are required. Despite all the difficulties, polyphenols should eventually find their place as candidates for creating on their basis not only antiviral drugs, but also for the prevention of cancer.

## 7 Chemosensitization of tumors with polyphenols

The search for effective methods of treatment and prevention of tumors, despite the successes achieved in recent decades, remains one of the most urgent tasks in medicine. Antitumor therapies (e.g., chemotherapy) use approaches based on induction of cell death by increasing the intracellular concentration of ROS (Wang et al., 2017). Since ROS are formed in cells not only as a result of the action of external physicochemical factors, but also in the processes of cellular metabolism, pharmacological correction of the redox properties of tumor cells is a promising approach to improve the effectiveness of antitumor therapy. In chemotherapy, drugs that enhance the production of ROS by cells are widely used. In recent decades, it has been shown that ROS generation is an important step in the process of induction of apoptosis of tumor cells by such widely used chemotherapeutic agents as cisplatin and doxorubicin (Li et al., 2020; Xia et al., 2020).

To increase the effectiveness of chemotherapeutic agents, approaches are proposed aimed at inducing oxidative stress in tumor cells. Therefore, pharmacological correction of the redox properties of tumor cells is a promising approach to improve the effectiveness of antitumor therapy. Currently, natural polyphenolic compounds are considered as preparations for the development of selective chemosensitizers. Due to the large number and variety of phenolic compounds, the antitumor properties of many of them have not been studied. In recent years, the regulatory properties of EGCG, the main catechin in green tea, resveratrol, one of the main polyphenols contained in the skin of grapes and red wines, and curcumin, the main curcuminoid that is part of the turmeric root, have been most actively studied (NavaneethaKrishnan et al., 2019). Polyphenols at low micromolar concentrations cause a protective antioxidant effect. At high concentrations, polyphenolic compounds exhibit pro-oxidant and cytotoxic properties. At the same concentrations in tumor and normal cells, polyphenols can induce oppositely directed effects (Oalđe Pavlović et al., 2021).

EGCG inhibits ROS generation in normal epithelial cells but induces ROS generation in tumor cells. In transformed cells, EGCG activates a mitochondria-mediated pathway of cell death, accompanied by the generation of ROS, a decrease in the transmembrane mitochondrial potential, and the release of apoptotic proteins (Min and Kwon, 2014). Similar results have been obtained in pancreatic cancer, lung cancer, colon cancer, and melanoma cell lines, as well as in breast cancer xenograft animal models (Gan et al., 2018; Khan and Mukhtar, 2018; Ravindran Menon et al., 2021; Romano and Martel, 2021). At concentrations of 5–20 μM, EGCG induced apoptosis only in melanoma cells, without any toxic effect on normal melanocytes (Ravindran Menon et al., 2021). At concentrations of 10–80 mg/ml, EGCG induced apoptosis in hepatocellular carcinoma cells, but not in the normal liver cells (Sojoodi et al., 2020). Sensitization of EGCG tumor cells to the action of a number of antitumor drugs has been shown in both *in vitro* and *in vivo* studies. EGCG enhances the effects of doxorubicin, 5-fluorouracil, cisplatin, trizenox (As2O3), bortezomib, and etoposide (Lecumberri et al., 2013; Almatroodi et al., 2020; Huang et al., 2020). Among the proposed mechanisms of tumor cell chemosensitization, the key role is played by redox modulation as a result of increased intracellular ROS production. For example, in ovarian cancer cells, EGCG increased cisplatin toxicity three to six fold, including in cisplatin-resistant cells (Zhou et al., 2014). However, the selectivity of the action of the compound in relation to tumor cells is not justified.

Numerous *in vitro* and *in vivo* studies have shown high antitumor activity of curcumin (see section “THE EFFECTIVENESS OF POLYPHENOLS”). It has been shown that the antitumor properties of curcumin are realized with the participation of ROS (Wan et al., 2019). It is assumed that curcumin causes ROS-induced decrease in transmembrane mitochondrial potential, resulting in activation of apoptosis (Wan et al., 2019). A number of studies have shown the ability of curcumin to activate autophagy through increased intracellular production of ROS (Tang et al., 2021). When autophagy is activated, mitochondria are the main source of ROS. At the same time, it is known that the functional relationship between apoptosis and autophagy is complex, that is, in some cases, autophagy is part of the cellular adaptation mechanism that protects cells from apoptosis, while in other conditions, autophagy can cause cell death or initiate apoptosis (Maiuri et al., 2007). It has recently been found that the molecular mechanism of sensitization of MCF-7 cells to the action of paclitaxel and adrenomycin by curcumin involves inhibition of the flap structure-specific endonuclease 1 (Fen1) endonuclease with the participation of ROS and NF-E2–related factor 2 (Nrf2) (Chen et al., 2014).

During therapy, resveratrol sensitizes tumor cells to the action of radiation therapy, cisplatin, doxorubicin, paclitaxel, and bortezamib (Fu et al., 2021; Ren et al., 2021). Synergy of action has been observed with the combined use of resveratrol and curcumin in relation to breast cancer, colon cancer, lung cancer and hepatocellular cancer (Niedzwiecki et al., 2016; Pavan et al., 2016; Arena et al., 2021). An increase in intracellular ROS production also plays a key role in the mechanism of cell sensitization under the action of resveratrol. It has been shown that resveratrol increases the sensitivity of pancreatic cancer cells as a result of Nrf2 activation and increased ROS production (Cheng et al., 2018). It is important to note that during radio- and chemotherapy, resveratrol protects normal cells from radiation damage and the toxic effects of chemotherapy drugs.

## 8 Conclusion and final remarks

Man consumes polyphenolic compounds throughout the evolutionary process, and these substances have been and remain a constantly present component of the internal environment of the body. Once in the body, they are involved in numerous processes of cell signaling, gene expression and various metabolic functions. Polyphenolic compounds, in particular flavonoids, are sometimes an inconspicuous but necessary link in the assembly and functioning of proteins, in the formation of biological membranes, and in the transmission of information in the cell. Being always available, they serve as helpers in many processes. It can be hoped that further research on polyphenols will make it possible to make many interesting discoveries, and the creation of artificial derivatives of flavonoids will make it possible to obtain new effective medicinal substances.

Targeting the stage of oncogenesis and development of tumors with polyphenolic compounds is a promising strategy for their use in both prevention and treatment (including increasing sensitivity to chemotherapy) of tumors. Data published in the literature that meet the requirements of evidence-based medicine confirm the beneficial effect of turmeric and rosemary with a high content of polyphenols, such as curcumin and carnazole acid, in pathological disorders in the body’s division processes, in particular, in oncological processes. Studies of the action of flavonoids show their ability to influence various life processes, both individual cells and the body as a whole. Although epidemiological studies of the relationship between the spread of cancer and the consumption of flavonoids have not yielded unambiguous results, in experimental conditions *in vitro* and *in vivo*, as well as in studies of volunteers, quite convincing evidence has been obtained of the promise of the use of certain flavonoids in the prevention and even in the treatment of tumors.
